# Predator Crown-of-Thorns Starfish (*Acanthaster planci*) Outbreak, Mass Mortality of Corals, and Cascading Effects on Reef Fish and Benthic Communities

**DOI:** 10.1371/journal.pone.0047363

**Published:** 2012-10-08

**Authors:** Mohsen Kayal, Julie Vercelloni, Thierry Lison de Loma, Pauline Bosserelle, Yannick Chancerelle, Sylvie Geoffroy, Céline Stievenart, François Michonneau, Lucie Penin, Serge Planes, Mehdi Adjeroud

**Affiliations:** 1 Laboratoire d'Excellence “CORAIL”, USR 3278 CNRS-EPHE, Centre de Recherches Insulaires et Observatoire de l′Environnement, Moorea, French Polynesia; 2 Laboratoire d'Excellence “CORAIL”, Institut de Recherche pour le Développement, U 227 COREUS 2, Nouméa, New Caledonia; 3 School of Mathematical Sciences, Queensland University of Technology, Brisbane, Queensland, Australia; 4 Australian Institute of Marine Science, Townsville, Queensland, Australia; 5 Department of Biology, University of Florida, Gainesville, Florida, United States of America; 6 Laboratoire d'Excellence “CORAIL”, Laboratoire d'Écologie Marine – ECOMAR, Université de La Réunion, Saint-Denis, La Réunion, France; The Australian National University, Australia

## Abstract

Outbreaks of the coral-killing seastar *Acanthaster planci* are intense disturbances that can decimate coral reefs. These events consist of the emergence of large swarms of the predatory seastar that feed on reef-building corals, often leading to widespread devastation of coral populations. While cyclic occurrences of such outbreaks are reported from many tropical reefs throughout the Indo-Pacific, their causes are hotly debated, and the spatio-temporal dynamics of the outbreaks and impacts to reef communities remain unclear. Based on observations of a recent event around the island of Moorea, French Polynesia, we show that *Acanthaster* outbreaks are methodic, slow-paced, and diffusive biological disturbances. *Acanthaster* outbreaks on insular reef systems like Moorea's appear to originate from restricted areas confined to the ocean-exposed base of reefs. Elevated *Acanthaster* densities then progressively spread to adjacent and shallower locations by migrations of seastars in aggregative waves that eventually affect the entire reef system. The directional migration across reefs appears to be a search for prey as reef portions affected by dense seastar aggregations are rapidly depleted of living corals and subsequently left behind. Coral decline on impacted reefs occurs by the sequential consumption of species in the order of *Acanthaster* feeding preferences. *Acanthaster* outbreaks thus result in predictable alteration of the coral community structure. The outbreak we report here is among the most intense and devastating ever reported. Using a hierarchical, multi-scale approach, we also show how sessile benthic communities and resident coral-feeding fish assemblages were subsequently affected by the decline of corals. By elucidating the processes involved in an *Acanthaster* outbreak, our study contributes to comprehending this widespread disturbance and should thus benefit targeted management actions for coral reef ecosystems.

## Introduction

**Figure 1:**
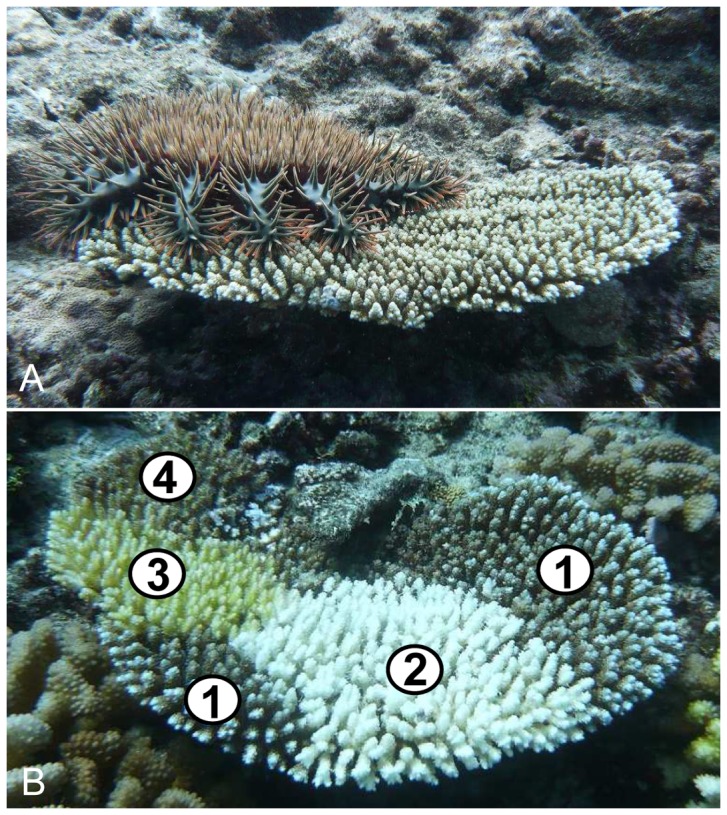
Photographs illustrating the outbreaking seastar *Acanthaster* and its feeding-scars as found on colonies preyed upon. (**A**) An *Acanthaster planci* observed on a living tabular coral from the genus *Acropora*. (**B**) A partially-killed coral from the genus *Acropora* bearing feeding-scars left by successive predation events by *Acanthaster*: **1**) live portion of the colony bearing the pigmented coral tissue, **2**) freshly killed portion of the colony deprived of its pigmented living tissue (<1 day post-predation), **3**) recently killed portion of the colony covered by early colonizing algae and cyanobacteria (∼10 days post-predation), **4**) dead portion of the colony killed long ago and covered by turf algae (>3 weeks post-predation). © Photos Mohsen Kayal.

**Figure 2:**
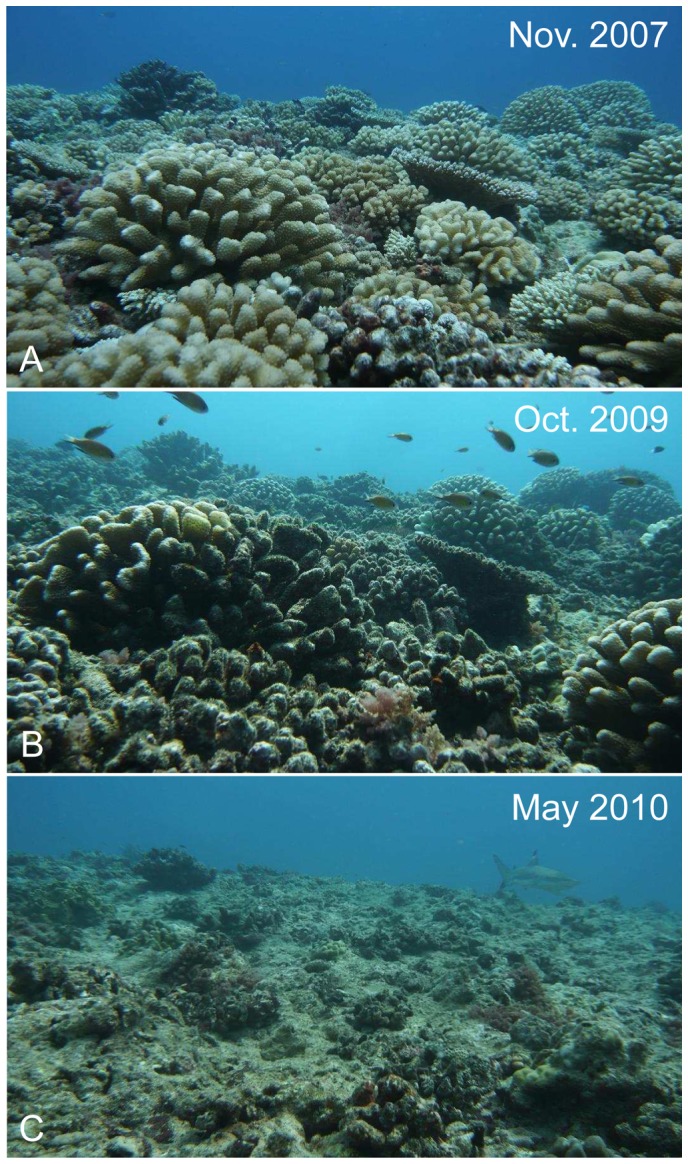
A portion of Moorean outer-reef (6 m-depth on the site Vaipahu) is shown through time. (**A**) Corals dominate the healthy reef (coral cover >40%). (**B**) Algae have colonized dead coral skeletons following severe predation by the seastar *Acanthaster* (∼10% coral cover). (**C**) Mostly dead and weakened coral skeletons were swept away by a cyclone occurring at the end of the seastar outbreak (Lison de Loma et al. *unpublished data*) and colonizing algae once again dominate the devastated reef (∼5% coral cover). © Photos Mohsen Kayal.

The crown-of-thorns seastar *Acanthaster planci* (Figure 1) is the major natural enemy of reef-building corals [1], [2]. This specialized coral-feeder is found on tropical reefs across the planet, except in the Atlantic Ocean. Populations of *Acanthaster* commonly display cyclic oscillations between extended periods of low-density with individuals scarcely distributed among large reef areas, and brief episodes of unsustainably high densities commonly termed ‘outbreaks’ [3]. These outbreaks are among the most destructive disturbances observed on tropical reefs [4], [5]. They result in mass mortalities of corals, sometimes annihilating populations, with typically second-order and long-term consequences on various communities [6]–[8]. Cascading effects of *Acanthaster* outbreaks usually spread to the entire reef ecosystem and commonly lead to increases in benthic algae, a loss of coral-feeding assemblages, an overall collapse of reef structural complexity, and a decline in biodiversity and productivity [6], [9]–[11] (see Figure 2 for illustrations). As a result, measures are often taken by local populations and management authorities to eradicate *Acanthaster* from reefs (e.g., [12]). However, such efforts often have limited success against the magnitude of outbreaks [6], [13]. *Acanthaster* outbreaks are increasingly documented [11], [14], yet these reports have mostly been restricted to short-term, sporadic observations (e.g., [5], [8], [15]). Until now, no study has quantitatively described the entire progression of an *Acanthaster* outbreak, including spatio-temporal dynamics of predator population and resultant impacts to the biological reef community. As a result, relatively little is known about the origins, development, or processes that influence the outcome of this disturbance [3], [7], [12], [16]–[26].

Here we describe an *Acanthaster* outbreak that occurred during the last decade in French Polynesia (South Pacific), a region where these disturbances occur with a periodicity of ∼20 years and, along with bleaching events and cyclones, are the major drivers of community dynamics on coral reefs [27]. Our study has specifically focused on the island of Moorea, where we tracked the distribution of *Acanthaster* aggregations and quantified their impacts on corals, other sessile communities, and resident coral-feeding fishes. In contrast to prior studies that were mostly restricted to reporting *a posteriori* observations of the consequences of *Acanthaster* on reefs, we quantitatively describe the processes leading to the community changes resulting from *Acanthaster* outbreaks.

## Methods

This study was approved and conducted as part of ongoing research of the Centre de Recherches Insulaires et Observatoire de l′Environnement (CRIOBE, USR 3278 CNRS-EPHE, LABEX “CORAIL”).

### Spatio-temporal scope of the study

**Figure 3:**
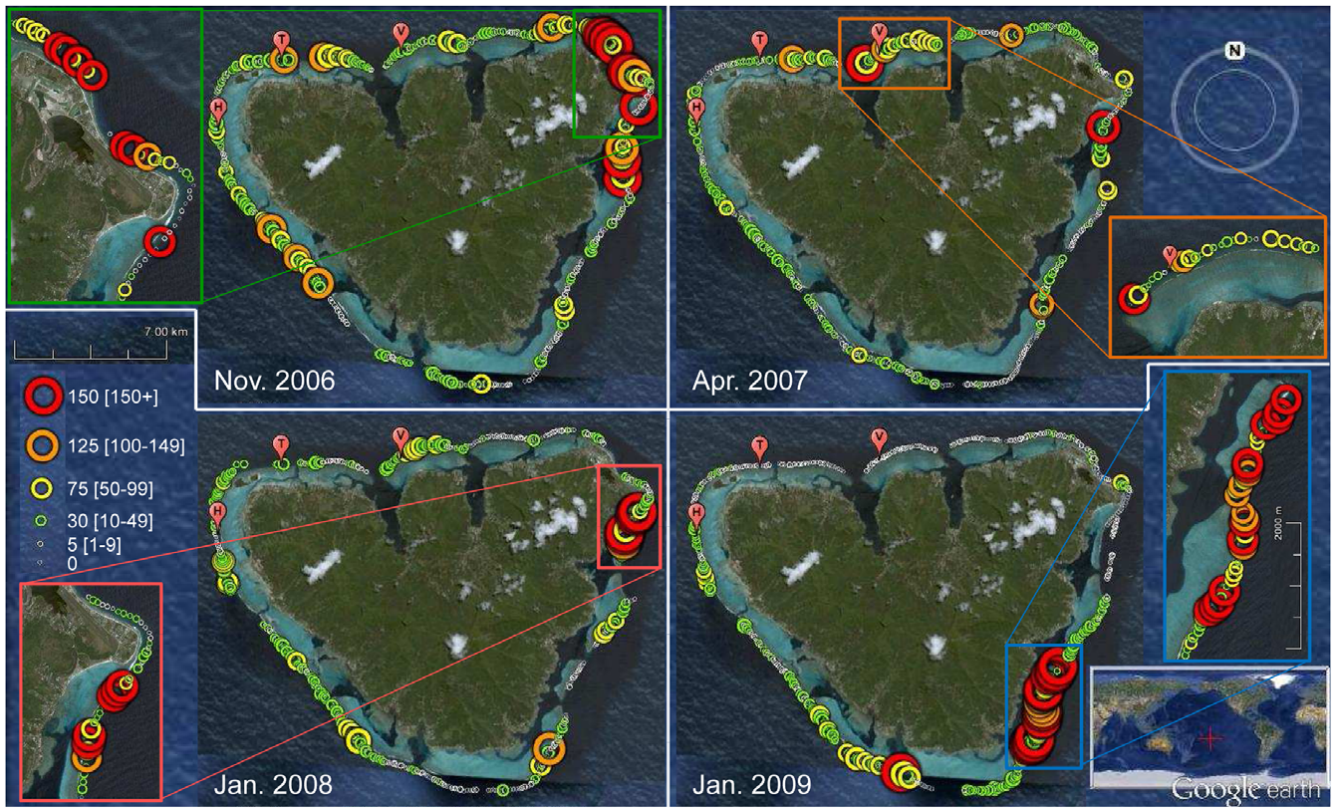
Distributions and densities of *Acanthaster* feeding-scars as observed around Moorea through time. The dimensions of the circles are proportional to the abundance of scars (n scars per 2 min-towing section), and a color code is used to distinguish different abundance classes (refer to the legend). A mean relation of 8.6±1.7 SE scars per individual seastar was estimated during the outbreak (see Table S1). The positions of the three reference sites where reef communities were surveyed are also displayed on the graphs: Haapiti (H), Tiahura (T), Vaipahu (V). Original satellite image from © Google Earth.

Starting in 2002, unusually elevated densities of *Acanthaster* were progressively reported from the different high volcanic islands of the Society Archipelago (Tahiti, Moorea, Huahine, Raiatea, Tahaa, Bora Bora, Maupiti) and then from the Australes (Rurutu) in French Polynesia. These islands are scattered over a broad geographical scale spreading 675 km north-south and 330 km east-west. Our study was conducted in Moorea (17°30′ S, 149°50′ W, see Figure 3), where long-term reef monitoring sites have been sampled for ∼40 years, and where the first *Acanthaster* aggregation was observed in 2003. Two complementary sampling approaches were used to quantify the dynamics of this outbreak and its consequences on reef communities. The first sampling approach was a periodic survey of various key functional assemblages among benthic and fish communities. These surveys were conducted at a small scale covering few hundred square meters of reef at nine reference reef locations, consisting of three water depths (6, 12, 18 m) at each of three sites (Vaipahu, Tiahura, Haapiti). The second sampling approach consisted of a yearly survey of seastar aggregations conducted at a large scale all around Moorea and its ∼100 km perimeter of reefs. The small-scale surveys were initiated between 2003 and 2005 depending on the different assemblages sampled (see Sampling section below), whereas the large-scale survey of seastars started in 2006, once *Acanthaster* aggregations were observed to spread to multiple sides of the island. Surveys were conducted until 2010, as long as remaining aggregations were observed. All the sampling was conducted using SCUBA on the outer reef slopes where, in Moorea as in other islands in French Polynesia, the highest coral biomass and the most diverse reef communities are concentrated [28]. This is also where *Acanthaster* aggregations were systematically first observed (refer to results of this study). Around Moorea, the outer reef habitat typically extends from the water surface at the crest of the barrier-reef where oceanic waves break, down to a depth of ∼35 m where sand plains begin. A peak in diversity and coral coverage is typically observed at the 10–20 m depth range [28], [29]. These outer reef habitats are exposed to the open ocean, undergo relatively little direct human pressure, and experience maximum exposure to natural disturbances [27]. By the end of the *Acanthaster* outbreak, Moorean reefs underwent the additional impacts of the tropical cyclone *Oli* (Lison de Loma et al. *unpublished data*) whose immediate effects were partially captured by the present study.

### Sampling strategy

Nine reef locations were surveyed on Moorean outer reefs in order to quantify community dynamics during the *Acanthaster* outbreak (see above). The composition of the sessile communities (i.e., relative coverage of coral populations and of other benthic components) was sampled in n = 10 random quadrats by recording the type of substrate beneath 81 points defined by a grid of 10 cm-mesh within the 1 m^2^ areas. As major reef corallivores, populations of the outbreaking seastar *Acanthaster* and resident coral-feeding butterflyfishes were surveyed in n = 3 replicate belt-transects (50×4 m) placed randomly along constant depth contours. These fishes belong to the genus *Chaetodon* which encompasses several specialized coral-feeding species whose populations are tightly associated with corals [1], [11]. The corallivorous representatives commonly encountered on Moorean outer reefs are *C. ornatissimus*, *C. reticulatus*, *C. trifascialis*, *C. lunulatus*, *C. auriga*, *C. lunula*, *C. pelewensis*, *C. ulietensis*, *C. unimaculatus*, *C. quadrimaculatus* [10], [30], [31]. To track the distributions and densities of *Acanthaster* aggregations at an island-scale around Moorea, we developed the SCUBA-tow technique, an adaptation of the manta-tow [8], [32] conducted using SCUBA [33]. The observer was positioned at 8 m-depth in the water column, ∼5 m above the reef substrate at a constant depth of ∼13 m, and towed by a boat all around the island at a speed of 4 knots (7.4 km h^−1^). In clear oceanic waters surrounding Moorea, this position of the observer allowed for a survey of the reef substrate in an ∼30 m-wide band in the 10–30 m depth range. Due to the cryptic character of *Acanthaster* [6], the density of seastars was estimated here by counting the number of characteristic feeding-scars found on colonies that were recently preyed upon (Figure 1). These white scars are denuded portions of the coral skeleton recently deprived of their pigmented living tissues, and can be used for tracking recent predation events [21], [34]. In the oligotrophic oceanic waters of French Polynesia, *Acanthaster* feeding-scars remain clearly visible for ∼3 weeks before being covered by colonising algae and other sessile organisms (see Figure 1). Counts were conducted in sections of 2 min of towing each covering a portion of ∼7,500 m^2^ of reef, and GPS coordinates were simultaneously recorded. The correspondence between the number of scars and the density of seastars was established by subsequent counts performed in transects on a restricted number of sites throughout the process of the outbreak (see Table S1).

### Statistical analysis

Variability in the coverage of benthic communities and in the density of butterflyfish assemblages was tested using three-way nested ANOVAs in which *Time* was nested within *Depth*, and *Depth* nested within *Site*. When significant differences were detected by ANOVA, Fisher's Least Significant Differences (LSD) *post-hoc* test was used to compare data among groups. Linear Mixed Models (LMMs) were used to test for correlations between the dynamics of the different reef communities: *Acanthaster*, corals, other sessile communities, and coral-feeding butterflyfishes. LMMs have the advantage of taking into account correlated observations, and were also used to examine how populations of different coral genera were affected during the decline of coral communities facing *Acanthaster*. This was performed by drawing a linear regression between the coverage of each coral genus (dependant variable *Genus cover*) and the coverage of the overall coral community (explicative covariable *Coral cover*) as quantified at our nine reference reef locations on four different years during the process of the outbreak (2005, 2008, 2009, 2010). For these regressions, autocorrelations were tested for the fixed effects of the covariable *Coral cover*, the random effects of the grouping factor *Reef location* (as the result of the interaction *Site* × *Depth*), and their interaction *Coral cover* × *Reef location*, and were taken into account in the calculation of parameters where significant [35], [36]. Before ANOVAs and LMMs, data were tested for normality and homoscedasticity, and were transformed when needed. *Acanthaster* and butterflyfish densities were log(*x*+1) transformed, and arcsin(√*x*) transformation was applied to percent-cover data. All statistics were computed in R version 2.12.0 (R Development Core Team 2008) complemented by the NLME package [36].

## Results

### *Acanthaster* outbreak

**Figure 4:**
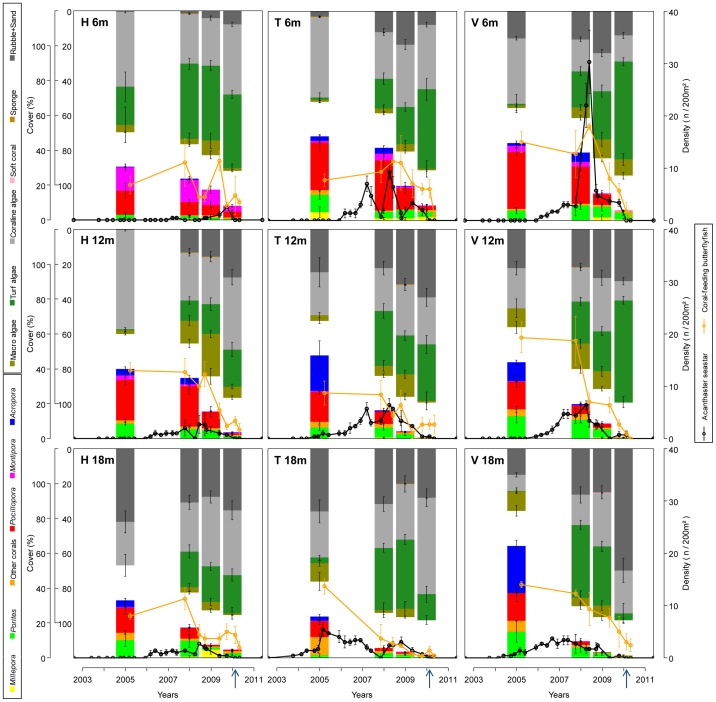
Dynamics of various communities surveyed at the reference reef locations. These nine locations consist of three sites (Haapiti: H, Tiahura: T, Vaipahu: V) × three water depths (6, 12, 18 m). Y-axes on the left indicate cover values (mean ± SE) of the sessile communities: reef-building corals and other benthic components. Y-axes on the right indicate densities (mean ± SE) of coral-predators: populations of the outbreaking seastar *Acanthaster* and butterflyfish assemblages. Arrows on the x-axes indicate the occurrence of the tropical cyclone *Oli*. Refer to Figure 5 for correlations between the dynamics of different communities. See Figure 2 for an illustration of the changes observed on reefs.

In Moorea, the first aggregation of *Acanthaster* was observed at 18 m-depth at the outer reef site Tiahura in October 2003 (Figure 4). The swarms of seastars affected the Vaipahu site also situated on the north shore of the island in May 2004. The Haapiti site on the west coast was affected in March 2006. SCUBA-tows performed in late 2006 showed that particularly high densities of *Acanthaster* feeding-scars (>150 scars per 2 min-towing section) were mostly concentrated on the north-eastern corner of Moorea, with elevated densities also found near Tiahura and on the central west side of the island (Figure 3). These scars were used as indirect evidences of recent *Acanthaster* predation on corals (see Figure 1), and an average ratio of 8.6±1.7 SE feeding-scars per seastar was calculated over the process of the outbreak (Table S1). Through consecutive years, these intense predation events spread over new reefs that were not yet affected, eventually affecting the entire coastline of Moorea. By 2009, most *Acanthaster* predation was concentrated near the southern tip of the island, with few feeding-scars observed on the formerly affected north shore (Figure 3).

Expanding waves of *Acanthaster* swarms developed similarly at all sites surveyed, consistently starting at the deepest locations of the outer reef and progressing upward with the migration of seastars along the reef-slope (Figure 4). This pattern varied among stations in terms of observed peak densities (min. 2.3±0.3 SE ind.200 m^−2^ or 11,500 ind.km^−2^ at Haapiti-6 m; max. 30.3±6.1 SE ind.200 m^−2^ or 151,650 ind.km^−2^ at Vaipahu-6 m) and residence times of predators (min. 30 months at Haapiti-6 m; max. 72 months at Tiahura-18 m), which, combined with the sequential time of arrival of seastar swarms at the different reef locations, resulted in complex spatio-temporal variability in the rate of predation on corals. This generated asynchronicity in the decline of corals among sites and depths (three-way nested ANOVA, factor *Time*(*Depth*(*Site*)), *p*<0.01; see Figure 4). The upward migration of aggregated seastars on the reef slope was observed in one (Vaipahu) or several (Tiahura and Haapiti) slow waves. By April 2010, densities of *Acanthaster* had fallen to zero on all surveyed reef locations, and no additional individuals were observed during subsequent surveys.

### Impacts on corals and other reef communities

**Figure 5:**
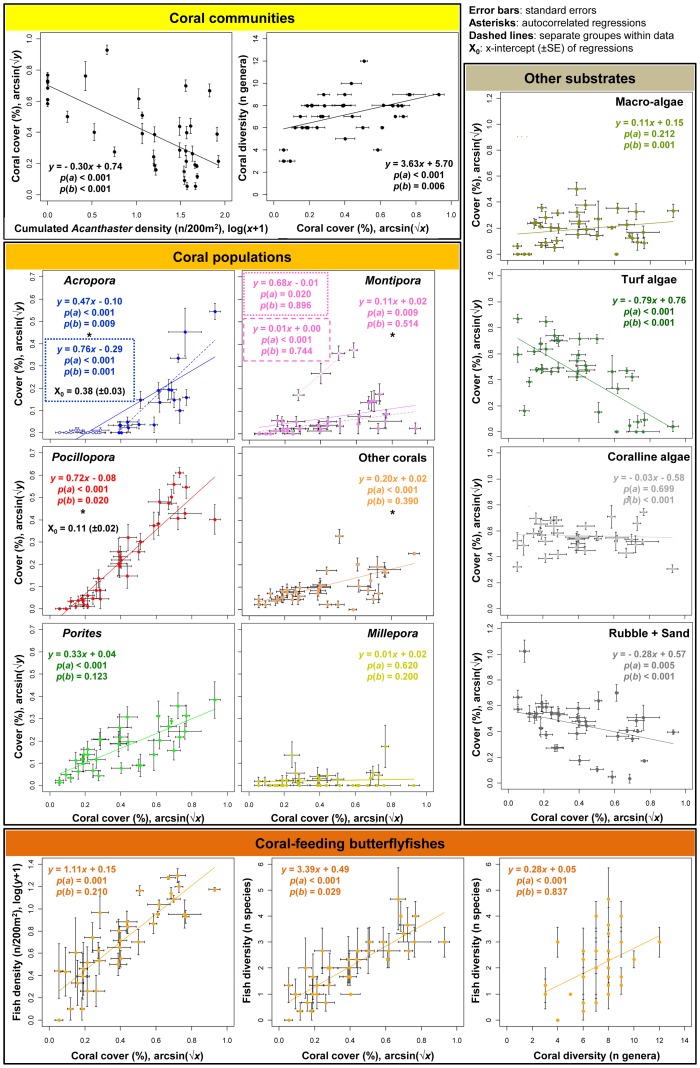
Correlations between the dynamics of different communities surveyed at the reference reef locations. Each point on the graphs (n = 36) represents the value (mean ± SE) of one sampling year (2005, 2008, 2009, 2010) on one site (Vaipahu, Tiahura, Haapiti) at one depth (6, 12, 18 m); refer to Figure 4. The equations and significance values (*p*) of the regression lines are given on the graphs (with *a*, the slope, and *b*, the intercept). The dynamics of the dominant coral genera are plotted against the dynamics of entire coral communities to quantify the sequential extirpation of populations by *Acanthaster*. An asterisk (*) indicates regressions for which significant autocorrelations were detected and taken into account in the calculation of parameters. For *Acropora*, data are split in two groups to distinguish samplings conducted during the decline phase (*Acropora*-cover >0.5%, solid dots, dashed regression line) and past the virtual extirpation of these populations from reefs (*Acropora*-cover <0.5%, empty circles, no regression line), the continuous line being the regression over the whole data set. For *Montipora*, data are split in two groups to distinguish samplings conducted at Haapiti-6 m where this taxon is predominating (empty circles, short-dashed regression line; see Figure 4) from the other stations (solid dots, long-dashed regression line), the continuous line being the regression over the whole data set. For coral genera whose populations were extirpated from the reef significantly earlier than the total coral community (i.e., *p*(*b*)<0.05), the x-intercept X_0_ (± SE) is also displayed.

*Acanthaster* predation resulted in a sharp collapse of coral populations and communities (Fisher's *p*<0.05 between consecutive samplings). This decline of corals progressively affected the different reef locations, as the seastars migrated through the reef system (Figures 4 and 5). The coral coverage decreased gradually from values mostly above 40% in 2005 to values often below 5% in 2010, sometimes <1% with the combined effects of the cyclone (at 12 m depth on the sites Tiahura and Vaipahu). Mass mortality of corals was accompanied by a decline in the diversity of coral assemblages, and was correlated with an increase in turf algae and dead-coral rubble and sand substrates (refer to the slopes of regressions in Figure 5, *p*(*a*)<0.05). In contrast, no significant trend was observed in the cover of macro-algae, coralline algae, soft corals, or sponges (*p*(*a*)>0.05, see Figure 4 and 5).

Coral genera were not equally impacted by *Acanthaster* predation, which resulted in a sequential extirpation of taxa from local communities (Figures 4 and 5). Branching and table-shaped species belonging to the genus *Acropora* were affected first and most heavily. Their populations declined abruptly and were extirpated from the reefs by the time coral cover fell below values of 13.7% (refer to the x-intercepts of regressions in Figure 5, arcsin(√X_0_) = 0.38±0.03 SE). The collapse of *Acropora* populations was followed by those of sub-branching *Pocillopora* (eliminated at 1.1% coral cover, arcsin(√X_0_) = 0.11±0.02 SE). Populations of encrusting *Montipora*, massive *Porites*, and other hard-coral assemblages also declined, showing a synchronized collapse with the entire coral communities (refer to the intercepts of regressions in Figure 5, *p*(*b*)>0.05). The calcifying hydrozoan *Millepora* was rarely preyed upon by the seastars, and its populations did not vary with the decline in live coral. The selective predation of *Acanthaster* on corals, combined with the sequential time of arrival of seastars at the different reef locations and the variability in the observed peak densities, generated high spatio-temporal heterogeneity in the structure of benthic communities. Within the seven years of the *Acanthaster* outbreak on Moorea (2003–2010), a gradual shift of the outer-reef system was observed, from a coral-dominated one, to an ecosystem where space was mainly occupied by turf algae, coralline algae, rubble, and sand (Figure 4). The community shift from corals to algae coincided with a shift in the composition of coral assemblages, from a state where branching genera *Acropora* and *Pocillopora* dominated in 2005, to one almost exclusively occupied by massive *Porites* in 2010.

Assemblages of coral-feeding butterflyfishes showed a tight correlation with corals in terms of size and diversity, resulting in a synchronous collapse of these populations with the mortality of corals (Figures 4 and 5). In February 2010, cyclone *Oli* generated ∼8 m waves that strongly affected the reef landscape by breaking and removing many live and dead coral skeletons at our study locations (see Figure 2). However, the potential effects of this second disturbance were partly diminished by the prior and ongoing occurrence of the *Acanthaster* outbreak which already had shown profound impacts on reef communities. The cyclone did not modify the pattern of decline in coral cover, increase in turf algae, and collapse of coral-feeding butterflyfishes as initiated since the beginning of the *Acanthaster* outbreak (Figure 4). After the passage of *Acanthaster* and cyclone, the once flourishing and polymorphic coral communities were mostly restricted to surviving fragments of massive *Porites* scattered among opportunistic algae (refer to Figure 2 for a synthetic illustration of the changes observed on the reef landscape).

## Discussion

Periodic outbreaks of the coral predator seastar *Acanthaster planci* constitute major disturbances to reef ecosystems in several regions throughout the Indo-Pacific [4], [5], [27]. Yet lack of observations of the development of these disturbances has restrained our knowledge of the ecological processes surrounding these events. During a particularly intense episode of *Acanthaster* outbreak around Moorea, French Polynesia, elevated *Acanthaster* densities spread from restricted source areas at the base of the northern outer reef and over several years propagated to the entire insular reef system. This propagation was based on a consecutive migration of *Acanthaster* aggregations toward unaffected adjacent and shallower reef locations. These waves of predatory seastars strongly impacted coral communities by decimating populations as encountered across reefs. The end of the outbreak coincided with the decimation of corals on the last affected reefs at the south of the island. This *Acanthaster* outbreak has been the most intense disturbance recorded on Moorean reefs since the establishment of scientific observations on this island about 40 years ago [27].

As observed in Moorea, *Acanthaster* outbreaks typically start at deeper locations at the base of reefs, where elevated cover in dead-coral rubble and coralline algae possibly favor settlement of the seastar larvae, provide shelter and food for the young juveniles that feed on coralline algae, and promote *Acanthaster* recruitment into adult coral-eating populations [6], [37]–[39]. The observed aggregative behavior of *Acanthaster* during outbreaks is thought to promote reproductive success [3], [6], [40], while resulting in the mass mortality of corals. *Acanthaster* has been shown to move relatively little in the presence of adequate food, however movements increase with higher densities of individuals and lower prey availability [6], [21]. During aggregations such as those recently observed in Moorea, the rapid local shortage in coral prey seems to engender an intensified foraging behaviour in *Acanthaster*. This behaviour is probably the major engine of the observed waves of migration, leading starving seastars to search for food in surrounding localities and spreading densities to unaffected reef locations. Such hunger-motivated directional movement of *Acanthaster* during outbreaks has already been suggested [38], [40], [41], and may explain the formation of high-density *Acanthaster* feeding fronts, as it is observed in other species (see [42], [43]). Interestingly, during the previous outbreak of this predator that was observed around Moorea in the early 1980′s, the first individuals of *Acanthaster* were also reported from the north shore of the island near the pass Taotoi [34], which is situated close to our site Tiahura where the first individuals were observed for the outbreak reported here. It remains unclear why this specific area would constitute a favourable settlement spot, nursery, or aggregating area for the development of *Acanthaster* outbreaks. The base of outer reefs on the north coast of Moorea accumulate relatively high concentrations of coral-rubble covered by coralline algae [29], which could favour recruitment of seastars [6], [39]. Yet further investigation is still needed to elucidate why specific reef locations constitute potential sources for *Acanthaster* infestations.

*Acanthaster* outbreaks typically induce considerable declines in corals, however the magnitude of decline is highly variable among outbreak events [5]. The coral communities around Moorea had shown relatively little fluctuations in size and structure since the turn of the millennium [27], and were drastically depleted by *Acanthaster* within a few months. Feeding preferences of *Acanthaster* consistently alter the structure of coral communities toward dominance by non-preferred species [1−3], [23], . Our observations show how this food selectivity results in a singular scheme of coral decline on affected reefs: *Acanthaster* hierarchically consumes preferred species and sequentially extirpates local populations. Density and residence time of predator *Acanthaster*, and local abundance and composition in prey corals, thus influence the magnitude of coral decline and the structure of surviving coral communities (see also [7]). In French Polynesia as on other reefs throughout the Indo-Pacific affected by periodic outbreaks, targeted attacks of *Acanthaster* on faster growing branching *Acropora* and *Pocillopora* populations result in episodic shifts of coral communities toward a temporary dominance by slower growing massive *Porites* [1], [2], [7], [15]. Thus, these natural disturbances constitute important historical drivers that shape the structure of coral communities in these regions.

Alterations of coral communities by *Acanthaster* are accompanied by subsequent changes in the demography of various reef species. As major competitors of corals, algae communities typically increase during outbreaks by colonizing the space released as corals die [16], [37], [38]. The recent increase in algae around Moorea has further been correlated to increases in herbivore assemblages, which in turn are preventing the development of macro-algal blooms as observed following coral mortality on other reefs [14], [44], [45]. Another re-emerging consequence of *Acanthaster* outbreaks is the collapse of resident corallivore assemblages that suffer from trophic limitations following the decimation of corals [9]–[11], [46]. Such shortage in food, rather than loss of refuges and habitat, was probably the main driver of the observed decline of coral-feeding butterlyfishes in Moorea. Indeed, *Acanthaster* predation does not alter the skeleton of corals and, over the short term, leaves the reef framework unaffected [1], [5], [11] (see Figures 1 and 2); yet these fishes showed a synchronous collapse with the decline of live coral during the outbreak, and were highly decimated on most reef areas before the physical alteration of their habitats by the cyclone. Similar *Acanthaster*-mediated loss of corallivores was also reported in decapod communities living within the branches of corals [26].

Following the relatively slow and diffusive devastation of corals by *Acanthaster*, the passage of the cyclone *Oli* resulted in an additional pulse disturbance that mostly affected the north shore of Moorea. This disturbance literally flattened the reef topography by breaking and removing the mostly-dead coral skeletons that remained after *Acanthaster* predation (Lison de Loma et al. *unpublished data*; see Figure 2 for illustrations). However, losses of reef structural complexity over the long-term have been attributed to *Acanthaster* outbreaks alone [9], [11]. While the cyclone showed limited immediate effects on surveyed benthic and fish species that were previously impacted by the seastar outbreak, the loss of physical structure on reefs likely had detrimental effects on other communities relying on corals as habitats and refuges [11], [26], [47], [48]. Furthermore, loss in reef structure will undoubtedly influence community regulation in the long-term [7], and may hinder the resilience of Moorean reefs. However, in contrast with a trend increasingly observed on reefs [4], [14], the strict regulation of algal communities to early-colonizing turf forms and the continuous flow of coral larvae among reefs in this region should lead to a progressive recovery of Moorean reefs, despite the tight recurrence of recent disturbances [27], [44], [45], [49].

### Conclusion

Our observations of an *Acanthaster* outbreak around Moorea coincide with records of a prior outbreak on this island [34] and with limited observations on other islands throughout French Polynesia also affected by the recent wave of infestations (*unpublished data*). Far from being unorganized and random events, outbreaks of the coral predator *Acanthaster planci* appear as ordered, relatively slow and diffusive biological disturbances. On the reefs surrounding the high volcanic islands of French Polynesia, these outbreaks were observed to originate from localized source areas situated at the base of outer-reef slopes, and to progressively spread to the entire reef systems by aggregative migrations of seastars. This pattern of propagation of *Acanthaster* from deeper parts of reefs toward unaffected locations also coincides with reports of previous outbreaks from other regions [38], [40], [41]. These seemingly hunger-driven assaults on corals decimate populations in a predictable sequence determined by feeding preferences consistently observed for *Acanthaster* [7], [15], [23], and their effects typically cascade down to many reef communities whose fates are directly or indirectly related to corals [9]–[11], [26], [40]. These findings improve our understanding of reef dynamics and have critical implications for management of coral ecosystems where *Acanthaster* is observed. We advocate the importance of monitoring the ocean-orientated bases of reefs, particularly those where coral-rubble and coralline algae are abundant. These measures could help detect *Acanthaster* outbreaks at the earliest stages and, when appropriate, improve the efficiency of control efforts.

## Supporting Information

Table S1 **Ratios of the density of feeding-scars to the number of predator seastar** ***Acanthaster*** **as observed in transect-counts on Moorean reefs during the outbreak.**
(PDF)
