# Supplementary material for: Predator Crown-of-Thorns Starfish (Acanthaster planci) Outbreak, Mass Mortality of Corals, and Cascading Effects on Reef Fish and Benthic Communities
Source: PLoS One. 2012 Oct 8;7(10):e47363. doi: 10.1371/journal.pone.0047363 (PMC3466260; doi:10.1371/journal.pone.0047363)
Supplement: Table S1 — Ratios of the density of feeding-scars to the number of predator seastar Acanthaster as observed in transect-counts on Moorean reefs during the outbreak. (PDF) [file pone.0047363.s001.pdf]

SUPPORTING INFORMATION

Table S1 Ratios of the density of feeding-scars to the number of predator seastar *Acanthaster* as observed in transect-counts on Moorean reefs during the outbreak. Transect-counts (n = 40) were conducted randomly through the process of the outbreak on reef locations where seastars were observed. Transects were led randomly along constant depth contours in the same portions of the reefs that were sampled by the SCUBA-tow technique (i.e., in the 10-30 m depth range on the outer reefs of Moorea). Because both *Acanthaster* density and coral coverage were varying through time and space over the span of the outbreak (refer to Figure 4 in the core of the manuscript), variability in the density of feeding-scars could result either from variations in abundance of predator seastars, or from differences in abundance of prey corals. Densities of *Acanthaster* feeding-scars were thus indirect evidences of the distribution and frequency of recent predation events, rather than quantitative measurements of the actual density of seastars on reefs. Results indicate a mean correspondence of 8.6 ±1.7 SE scars counted per individual *Acanthaster*, meaning that seastars were on average feeding 8-9 times per period of ~ 3 weeks, time laps during which the feeding-scars were remaining clearly visible following recent predation (see Figure 1 in the core of the manuscript for illustrations).

Date	Site	Transect size	n Scar	n *Acanthaster*	n Scar / n *Acanthaster*
Nov. 2006	Opunohu West	$250m^2$ (50m x 5m)	68	7	9.71
Nov. 2006	Opunohu West	$250m^2$ (50m x 5m)	41	9	4.56
Nov. 2006	Opunohu West	$250m^2$ (50m x 5m)	26	2	13
Nov. 2006	Opunohu East	$250m^2$ (50m x 5m)	77	7	11
Nov. 2006	Opunohu East	$250m^2$ (50m x 5m)	5	1	5
Nov. 2006	Opunohu East	$250m^2$ (50m x 5m)	5	2	2.5
Nov. 2006	Opunohu East	$250m^2$ (50m x 5m)	10	1	10
Nov. 2006	Vaiare	$250m^2$ (50m x 5m)	53	6	8.83
Nov. 2006	Vaiare	$250m^2$ (50m x 5m)	52	4	13
Nov. 2006	Haapiti Taotaha	$250m^2$ (50m x 5m)	62	7	8.86
Nov. 2006	Haapiti Taotaha	$250m^2$ (50m x 5m)	34	3	11.33
Apr. 2007	Vaipahu	$350m^2$ (25m x 14m)	75	7	10.71
Apr. 2007	Tiahura	$350m^2$ (25m x 14m)	7	1	7
Apr. 2007	Haapiti	$350m^2$ (25m x 14m)	26	3	8.67
Jan. 2008	Vaipahu	$250m^2$ (50m x 5m)	16	1	16
Jan. 2008	Vaipahu	$250m^2$ (50m x 5m)	3	1	3
Jan. 2008	Vaipahu	$250m^2$ (50m x 5m)	12	3	4
Jan. 2008	Haapiti	$250m^2$ (50m x 5m)	55	1	55
Jan. 2008	Haapiti	$250m^2$ (50m x 5m)	73	3	24.33
Jan. 2008	Haapiti	$250m^2$ (50m x 5m)	69	2	34.5
Jan. 2008	Vaiare	$250m^2$ (50m x 5m)	42	8	5.25
Jan. 2008	Vaiare	$250m^2$ (50m x 5m)	66	6	11
Jan. 2008	Vaiare	$250m^2$ (50m x 5m)	23	1	23
Jan. 2008	Vaiare	$250m^2$ (50m x 5m)	6	1	6
Jan. 2008	Vaiare	$250m^2$ (50m x 5m)	22	2	11
Jan. 2009	Vaipahu	$250m^2$ (50m x 5m)	6	3	2
Jan. 2009	Vaipahu	$250m^2$ (50m x 5m)	15	2	7.5
Jan. 2009	Vaipahu	$250m^2$ (50m x 5m)	0	1	0
Jan. 2009	Vaipahu	$250m^2$ (50m x 5m)	1	1	1
Jan. 2009	Vaipahu	$250m^2$ (50m x 5m)	4	1	4
Jan. 2009	Vaipahu	$250m^2$ (50m x 5m)	0	1	0
Jan. 2009	Vaipahu	$250m^2$ (50m x 5m)	0	4	0
Jan. 2009	Vaipahu	$250m^2$ (50m x 5m)	0	1	0
Jan. 2009	Haapiti	$250m^2$ (50m x 5m)	0	3	0
Jan. 2009	Haapiti	$250m^2$ (50m x 5m)	1	5	0.2
Jan. 2009	Haapiti	$250m^2$ (50m x 5m)	27	6	4.5
Jan. 2009	Haapiti	$250m^2$ (50m x 5m)	0	4	0
Jan. 2009	Haapiti	$250m^2$ (50m x 5m)	0	3	0
Jan. 2009	Vaiare	$250m^2$ (50m x 5m)	0	1	0
Jan. 2009	Vaiare	$250m^2$ (50m x 5m)	8	1	8
				**Mean**	**8.61**
				**SE**	**1.67**
